# A Comparison of Failure Loads for Polycrystalline Zirconia Ceramics with Varying Amounts of Yttria, Glass-Ceramics and Polymers in Two Different Test Conditions

**DOI:** 10.3390/polym15234506

**Published:** 2023-11-23

**Authors:** Tariq F. Alghazzawi

**Affiliations:** 1 Department of Substitutive Dental Sciences, Taibah University, Madinah 42353, Saudi Arabia; tghazzawi@taibahu.edu.sa; 2 Department of Mechanical and Materials Engineering, The University of Alabama at Birmingham, Birmingham, AL 35294, USA

**Keywords:** zirconia, glass-ceramic, crown, failure load, yttria concentration

## Abstract

It is unclear how zirconia dental crowns with different yttria compositions will perform clinically, and how they will compare with crowns made of glass-ceramics and polymers. The present objective was to determine failure loads of crowns and discs made of glass ceramics or polymers as compared to yttria-partially stabilized zirconia (Y-PSZ) crowns and discs with varying yttria concentrations. Crowns of zirconia (Cercon XT, Katana UTML, BruxZir Anterior), glass ceramic (Celtra press, IPS e.max press, Lisi press), and polymeric materials (Trilor, Juvora, Pekkton) were fabricated and cemented to epoxy abutments. The total number of specimens was 135 for crowns and 135 for discs (n = 15 specimens per material type and design). A universal testing machine was used to perform compressive loading of crowns/discs to failure with a steel piston along the longitudinal axis of the abutments. Energy dispersive spectroscopy (EDS) was used to identify the yttria concentration for each zirconia brand. The data were analyzed using generalized linear models and regression analyses. The results revealed significant differences (*p* < 0.05) in mean failure loads for different crown materials: Trilor (6811 ± 960 N) > Juvora (5215 ± 151 N) > Cercon (4260 ± 520 N) = BruxZir (4186 ± 269 N) = e.max (3981 ± 384 N) > Katana (3195 ± 350 N) = Lisi (3173 ± 234 N) = Pekkton (3105 ± 398 N) > Celtra (2696 ± 393 N). The general linear model revealed significant differences (*p* < 0.05) in mean failure loads when comparing the different materials for the discs, i.e., Trilor (5456 ± 1748 N) > Juvora (4274 ± 869 N) > Pekkton (3771 ± 294 N) > Katana (2859 ± 527 N) > Cercon (2319 ± 342 N) = BuxZir (2250 ± 515 N) = e.max (2303 ± 721 N) = Lisi (2333 ± 535 N) > Celtra (1965 ± 659 N). EDS showed that the zirconia materials contained yttria at different concentrations (BruxZir = 5Y-PSZ, Cercon = 4Y-PSZ, Katana = 3Y-PSZ). The yttria concentration had a significant effect on the failure load of the Katana (3Y-PSZ) crowns, which revealed lower failure loads than the Cercon (4Y-PSZ) and BruxZir (5Y-PSZ) crowns, whose failure loads were comparable or higher than e.max glass ceramic. The failure load of the trilayer disc specimens did not correlate with the failure load of the respective crown specimens for the zirconia, glass-ceramic and polymeric materials.

## 1. Introduction

Yttria-stabilized tetragonal zirconia polycrystal (Y-TZP) materials are a possible replacement for metal ceramic restorations because of their good mechanical properties [1,2]. However, the translucency of pure tetragonal Y-TZP has limited its use in anterior regions, where the color is affected by the resin cement and dentin underneath the crown [3,4]. Veneered porcelain has been applied on tetragonal Y-TZP copings to mask the opacity of the zirconia. The literature [5] reported that the chipping of veneer porcelain was a concern; therefore, tetragonal Y-TZP crowns with staining were considered an option for treatment [6,7]. With the development of newer engineering materials, part of the tetragonal phase has been replaced with the cubic phase to yield yttria-partially stabilized zirconia (Y-PSZ) with a cubic phase that improves optical properties to be comparable with glass ceramic restorations [8]. The mechanical properties of zirconia restorations can be attributed to the type, and amount of stabilizing oxide [9,10,11,12,13,14,15], surface treatment [16,17,18], sintering conditions [19], ceramic thickness [15,16], zirconia phase [20,21], and translucency [22]. It has been shown that decreasing the amount of yttria strengthens zirconia [15], while increasing the yttria concentration increases its translucency [9], thus creating a trade-off between these desirable properties.

In comparison, the mechanical properties of glass ceramic restorations are affected by the material type [23,24], cement thickness [25], surface polishing [26], glazing [27], thickness [28,29], and adhesive cementation [30]. Polymeric materials may provide another alternative to ceramic materials because they show adequate strengths and are more ductile. Aldhuwayhi et al. [31] reported that polyetheretherketone (PEEK) posterior tooth crowns had a greater fracture resistance and less catastrophic failures with some plastic deformation as compared to IPS e.max^®^CAD crowns. The strengths of high-performance PEEK and polyetherketoneketone (PEKK) depend on their functional groups and molecular bonds, while newly developed fiber-reinforced composite resins are strengthened by nanotubes and fibers of various types [32].

“Crunch the crown” (CTC) testing, where the crown is attached to an abutment with resin cement and loaded with a steel piston, has been used to simulate clinical conditions and to evaluate the strength of the restoring materials [33,34]. It has been demonstrated that the abutment must be fabricated with a modulus of elasticity similar to dentin to achieve realistic results; however, the CTC test protocol has not yet been standardized.

Alternative in vitro tests have been proposed in an effort better simulate the clinical behavior of dental restorations [30,35], including a trilayer model in the shape of a disc (5 mm thickness, 10 mm diameter, to approximate the surface of molars). The first layer simulates dental restoration and has a thickness of 1.5 mm. The second layer is fabricated from epoxy resin to simulate dentin and has a thickness of 2.5 mm. The third layer simulates pulp with a thickness of 1.5 mm and is fabricated from steel. It is not known whether results from this testing protocol will correlate with those of traditional CTC tests and is, therefore, worthy of investigation.

It is unclear how the mechanical properties of zirconia dental crowns with different yttria compositions will vary, and how they will compare with crowns made of glass-ceramics and polymers. The objectives of the present study were to determine the failure loads of crowns/discs made of glass ceramics or polymers and to compare them with yttria-partially stabilized zirconia (Y-PSZ) crowns/discs with varying yttria concentrations. Author hypothesized that the failure loads of zirconia with a lower yttria concentration would be higher than those for the glass-ceramics and polymers, and the failure load of crown specimens would correlate with those of disc-shaped specimens.

## 2. Materials and Methods

Crown specimens were created from a monolithic crown-on-abutment design containing the various test materials: zirconia (BruxZir Anterior, Katana UTML, Cercon XT); glass ceramic (Celtra Press, IPS e.max Press, Lisi Press); and polymeric dental materials (Trilor, Juvora, Pekkton). The identifier, manufacturer, color, class, modulus of elasticity, and Poisson’s ratio of the test materials are listed in Table 1 according to the manufacturer data. The total number of crown specimens was 135 (n = 15 specimens per material type and design). An ivorine maxillary 1st molar tooth (Model #R861; Columbia Dentoform Corp, Long Island City, NY, USA) was prepared with 1.0 mm rounded shoulder around the entire circumference, an occlusogingival height of 4 mm, 12° total convergence angle, and occlusal reduction of 1.5 mm in the area of the occlusal surface [36], as a model for the crown specimens, and scanned with a 3-D scanner (Model D2000, 3Shape A/S, Copenhagen, Denmark).

The zirconia and glass ceramic crowns were designed with a cement gap of 35 µm and an extra cement gap of 65 µm (the cement gap is the amount of offset in the area of the margin line while the extra cement gap is the amount of offset in the upper part of the interface). In contrast, the polymeric crowns were designed with a cement gap of 75 µm and an extra cement gap of 120 µm (according to the manufacturer’s instructions). The zirconia and polymeric crowns were fabricated using a Wieland Mini milling machine (Wieland Dental + Technik GmbH & Co. KG, Pforzheim, Germany) from prefabricated discs and the zirconia specimens were sintered according to the manufacturer instructions, in which the multiple layers of different colors were retained the same for all specimens. Glass-ceramic specimens (Celtra Press, IPS e.max Press, Lisi Press) were fabricated by digital designation of wax patterns, printed (Cara 2.0 printer, Kulzer, South Bend, IN, USA), and then heat pressed with glass-ceramic ingots of different materials (Celtra Press, IPS e.max Press, Lisi Press). All crowns were fabricated with the occlusal thickness of 0.8 mm at the central fossa. The supporting epoxy resin abutments for the crowns were fabricated by duplicating the molar tooth and pouring it into epoxy resin to form a resin abutment with a height of 8 mm which represents the height from the highest point of the prepared molar tooth to lowest point to the roots, as described by Schriwer et al. [36].

The “intaglio” surface treatments of the zirconia, glass-ceramic, and polymeric crowns are described in Table 2 and are consistent with previous studies [30,35]. After the surface treatments were completed, the Clearfil ^TM^ ceramic primer plus (Kuraray America, Inc., New York, NY, USA) was applied and air dried, and then the crowns were bonded to the corresponding resin abutments using Panavia V5 (Kuraray America, Inc., New York, NY, USA). A load of 50 N was applied to the specimens during the cementation process to obtain an even cement thickness. This was followed by the removal of excess cement and subsequent light curing using a VALO LED curing-light (Ultradent Products, Inc., South Jordan, UT, USA) for 30 s in 5 directions [30,35]. The cemented crown/abutment specimens were stored in 37 °C water for 24 h before testing.

**Figure 1:**
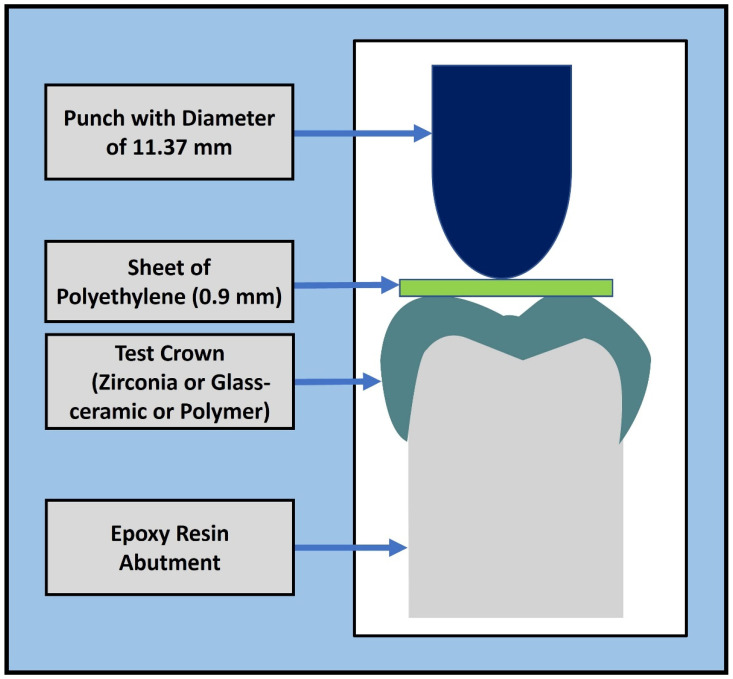
Schematic of the loading protocol for the crown with abutment. A polyethylene sheet with a thickness of 0.9 mm was used between the steel piston and occlusal surface of the crown to reduce contact stresses.

In the CTC tests, the crowns were compressed with an axial load applied through a steel piston (diameter of 11.37 mm), as shown in Figure 1 [36]. A polyethylene sheet with a thickness of 0.9 mm was used between the steel piston and occlusal surface of the crown to reduce contact stresses. The sheet was used to reduce the contact stresses between the crown and the steel piston, in a method similar to Schriwer et al. [36]. The displacement-control tests were conducted using a universal testing machine (MTS Model 858, Mini-Bionix, Eden Prairie, MN, USA) at 0.5 mm per minute until failure. The failure load was recorded as the maximum force attained during each test.

**Figure 2:**
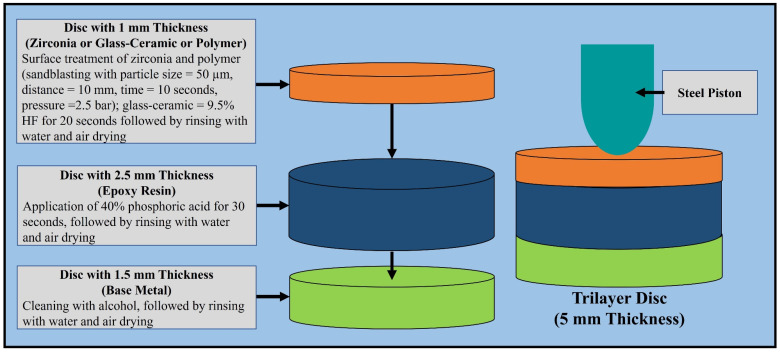
The protocol for the surface treatment of the three monolayer discs where the surface materials (zirconia/glass-ceramic/polymer) were attached to epoxy resin and steel rings and loaded with a steel piston.

Trilayer disc specimens (Figure 2) were created as described by de Kok et al. [30]. The ceramic and polymeric discs were fabricated using a Wieland Mini milling machine. The epoxy resin disc was milled from a prefabricated 2.5 mm thick epoxy plate. The steel rings were milled with the following specifications: inner diameter = 6.5 mm, outer diameter = 10.0 mm, and thickness = 1.5 mm. The total number of disc specimens used was 135 (n = 15 specimens per material type and design). The ceramic and polymeric discs were bonded to the epoxy discs using Panavia V5 (Kuraray America, Inc.). The two-layer discs were then bonded to the steel rings using Clearfil SE Bond (Kuraray America, Inc.) [35]. All trilayer disc specimens were stored in 37 °C water for 24 h before testing. The quasi-static loading test followed the same protocol as de Kok et al. [30], and was performed with a hemisphere (4.9 mm diameter) centered on the top surface of the trilayer disc specimen and compressed using the material testing machine at 0.5 mm/min until failure.

The fracture surfaces of the zirconia specimens were sputter coated with gold palladium, and scanning electron micrographs (Quanta FEG 650 scanning electron microscope, Lafayette, IN, USA) were obtained using high-vacuum mode with an accelerating voltage of 30 kV. Energy dispersive spectroscopy (EDS) was then used in a blind matter to identify the concentration of yttria for each zirconia brand over multiple areas, for which an average was calculated as documented in the literature [9].

Comparative analyses for failure loads were performed for the crown and disc specimens among the 9 materials and 3 classes (zirconia, glass ceramics, and polymers). Homogeneity of variance was tested using Levene’s test, and normality was tested with the Kolmogorov–Smirnov test. The rank transformation was used to improve the distribution characteristics. Analysis was subsequently carried out with SPSS v23 (IBM CORP, Armonk, NY, USA) using a generalized linear model with a robust estimator on both crown and disc designs. Comparisons between the materials were carried out with the sequential Bonferroni method for multiple comparisons. Linear regression analysis was used to test for correlation between the mean failure load values of the crowns and discs for each class of material (zirconia, glass ceramic, polymer). Pearson’s correlation coefficient (R-squared) revealed the goodness of fit, while *p* values associated with each regression were determined; *p* < 0.05 was considered significant.

## 3. Results

**Figure 3:**
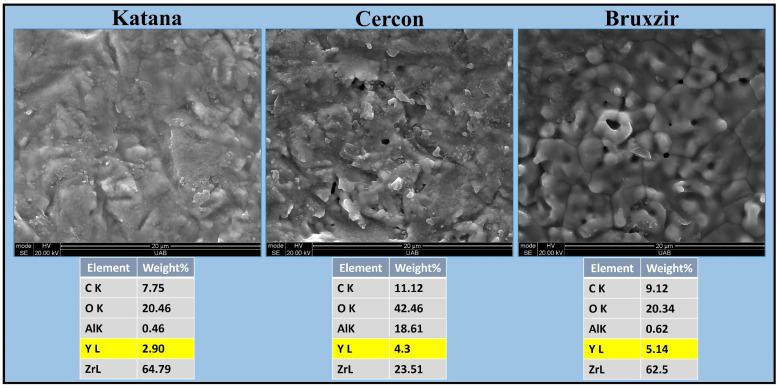
SEM images (12,000× SE) of fractured surfaces and element analysis (EDS) for the three zirconia materials. Zirconia brands had different yttria concentrations (KAT = 3Y-PSZ, CER = 4Y-PSZ, BRU = 5Y-PSZ). SEM = scanning electron microscope, KAT = Katana UTML, CER = Cercon XT, BRU = BruxZir Anterior, C = Carbon, O = Oxygen, Al = Aluminum, Y = Yttrium, Zr = Zirconium.

The fractured zirconia surfaces were fairly heterogeneous, with grains at different surface levels and diffuse boundaries. Backscatter SEM showed that all zirconia materials had an yttria stabilizing oxide with different concentrations (KAT = 3Y-PSZ, CER = 4Y-PSZ, BRU = 5Y-PSZ), as illustrated in Figure 3. Additional SEM images of the KAT, CER and BRU failure surfaces may be viewed in our previous manuscript [37].

**Figure 4:**
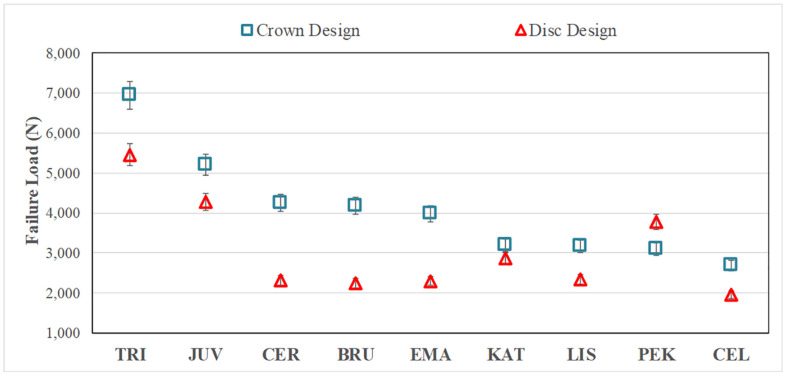
Failure loads (mean + standard deviation, in Newtons) for crowns and discs. Except for PEK, the crown design specimens had a higher average failure load than the disc design specimens. The general linear model revealed significant differences (*p* < 0.05) in mean failure loads when comparing the different materials for the crowns, i.e., TRI > JUV > CER = BRU = EMA > KAT = LIS = PEK > CEL, and for the discs, i.e., TRI > JUV > PEK > KAT > CER = BRU = EMA = LIS > CEL. TRI = Trilor, JUV = Juvora, CER = Cercon XT, BRU = BruxZir Anterior, EMA = IPS e.max Press, KAT = Katana UTML, LIS = Lisi Press, PEK = Pekkton, CEL = Celtra Press.

TRI and JUV (polymer) crowns yielded the highest mean failure loads, followed by CER and BRU (zirconia). There were no significant differences (*p* = 0.072) between the mean failure loads (Figure 4) comparing the CER (4260 ± 520 N) = BRU (4186 ± 269 N) = EMA (3981 ± 384 N) crowns; however, each was significantly higher than KAT (3195 ± 350 N). The specimens for zirconia, glass-ceramic, and PEK crowns failed by brittle fracture, whereas TRI and JUV materials failed by excessive deformation.

The layered disc results showed similar trends as the crowns (Figure 4), but with lower failure loads (roughly 20–60%), except for the KAT discs, which failed at values close to the crowns and the PEK discs, which failed at higher loads than the respective CTC tests. The general linear model revealed significant differences (*p* < 0.05) in mean failure loads when comparing the different materials for the discs, i.e., TRI (5456 ± 1748 N) > JUV (4274 ± 869 N) > PEK (3771 ± 294 N) > KAT (2859 ± 527 N) > CER (2319 ± 342 N) = BRU (2250 ± 515 N) = EMA (2303 ± 721 N) = LIS (2333 ± 535 N) > CEL (1965 ± 659 N).

**Figure 5:**
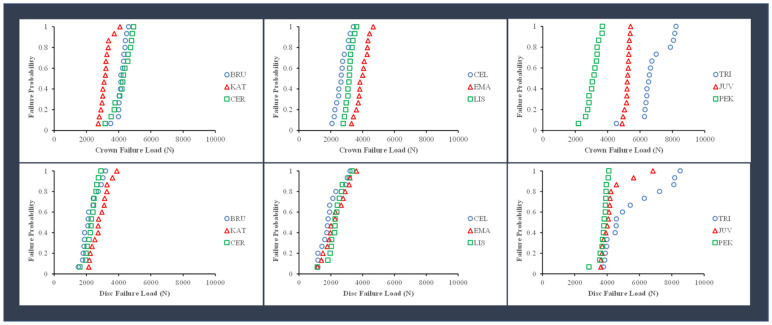
Failure probability for the failure load of the disc and the crown specimens for zirconia, glass-ceramics, and polymers. KAT, CEL, LIS, and PEK had higher failure probabilities for the crowns. For the discs, the failure probability of the materials is almost the same for each group. TRI = Trilor, JUV = Juvora, CER = Cercon XT, BRU = BruxZir Anterior, EMA = IPS e.max Press, KAT = Katana UTML, LIS = Lisi Press, PEK = Pekkton, CEL = Celtra Press.

The probability of failure for the different material groups, calculated as the value of the failure load divided by the number of specimens (15), is shown in Figure 5. Among the zirconia crown specimens, there was a higher risk of fracture at lower loads for KAT crowns than for BRU and CER crowns. Among the glass-ceramic crown specimens, there was a higher fracture risk at smaller loads for CEL and LIS crowns than for EMA crowns. Among the polymer crown specimens, there was a higher risk of fracture for the PEK crowns at smaller loads than for JUV and TRI crowns.

**Figure 6:**
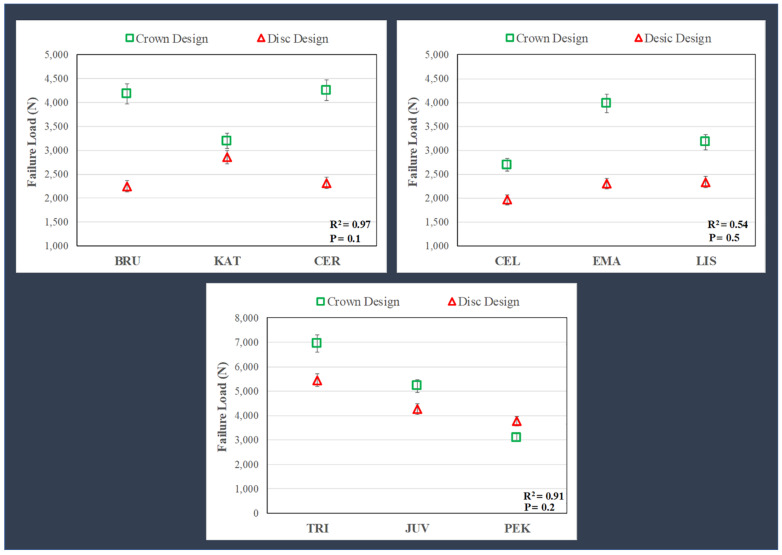
The correlation between the crowns and discs for three different groups of materials (zirconia, glass ceramics, polymers). Linear regression showed no correlations (*p* > 0.05) between failure load for the crowns and discs, as shown here by the inconsistent differences in values and trends for the crowns and discs within each group of materials. TRI = Trilor, JUV = Juvora, CER = Cercon XT, BRU = BruxZir Anterior, EMA = IPS e.max Press, KAT = Katana UTML, LIS = Lisi Press, PEK = Pekkton, CEL = Celtra Press.

As shown in Figure 4, the crown specimens for zirconia, glass-ceramics and polymeric materials produced higher mean failure loads than the layered disc specimens, except for the PEK material. The relationships between the average failure loads for the crown and disc specimens for the three different groups of materials are shown in Figure 6. There were no significant correlations (*p* > 0.05) between the failure loads for the crown and disc specimens, which was determined by linear regression for the material groups. As shown in Figure 5, there was a higher fracture risk at lower loads for the discs than for the crowns. The failure probability curves were more distinct for crown specimens than disc specimens.

## 4. Discussion

The variations in the composition (yttria concentration) of zirconia were not discernable in the data provided by the manufacturers. Therefore, EDS was performed in the present study to quantify yttria concentration for each zirconia brand. The CER (4Y-PSZ) and BRU (5Y-PSZ) produced similar mean failure loads (4260 N and 4186 N, respectively), statistically higher than those for KAT (3Y-PSZ = 3195 N). Thus, the hypothesis that yttria concentration would correlate with crown failure load was only partially supported.

Mayinger et al. [11] reported that the 4Y-TZP (Ceramill Zolid HT+, Amann Girrbach) material presented CTC failure loads similar to 3Y-TZP (Ceramill Zolid, Amann Girrbach) for a three-unit fixed partial denture. Kongkiatkamon et al. [22] reported that the failure load of Cercon XT = 3317.76 ± 199.80 N, lower than those found presently, where CER failure loads averaged 4260 ± 520 N. Elsayed et al. [9] reported that increasing the concentration of yttria (D Bio ZX2; 3Y-TZP > DD cubeX2 HS; 4Y-TZP > DD cubeX2; 5Y-TZP) in an attempt to improve the optical properties can reduce the mechanical properties. Abdulmajeed et al. [15] reported that lowering the yttria concentration increased the failure load of zirconia (Katana UTML; 5Y-PSZ > Katana UTML; 4Y-PSZ > Katana HT; 3Y-PSZ). These results, along with present findings, demonstrate that clinicians should carefully choose cubic zirconia to increase the translucency since the yttria concentration affects the resulting mechanical properties.

The results were contradictory for the failure loads of the zirconia specimens comparing the two different specimen geometries. The 3Y-PSZ presented much higher strengths than 4Y-PSZ and 5Y-PSZ for layered disc geometry, as documented in the literature [15]. Surprisingly, the results were the opposite for the crown specimens, where the 4Y-PSZ and 5Y-PSZ specimens revealed much higher failure loads than 3Y-PSZ. Possible reasons for this discrepancy include the role of crown thickness [15,16], surface treatment [16,17,18], and cement type [38]. There are no ISO and ASTM standards for crown testing, in terms of cement type/thickness, crown thickness, or internal surface treatment. Our experiments tested crowns from three classes of materials, where the cement thickness and internal surface treatment were different based on manufacturer’s recommendations for each material. The present values of CTC failure loads may not, therefore, be directly comparable to values obtained in previous studies.

In the present study, EMA (lithium disilicate) yielded an average crown failure load similar to those of CER (4Y-PSZ) and BRU (5Y-PSZ), but higher than KAT (3Y-PSZ). Choo et al. [14] reported that 5Y-PSZ (Katana UTML) had a lower failure load than lithium disilicates (e.max CAD) for monolithic anterior crowns. Lawson et al. [10] reported that there was no significant difference in failure load between 5Y-PSZ (Lava Esthetic) and lithium disilicate (e.max CAD), whereas 3Y-PSZ (Lava Plus) had a higher failure load for crowns with 0.8 mm uniform thickness. Yan et al. [23] reported that lithium disilicate had load-bearing properties similar to those of 4Y-PSZ but much better than those of 5Y-PSZ.

The EMA glass ceramic specimens revealed a higher average failure load than CEL and LIS. This result was likely due to the different crystalline types (LIS, EMA = lithium disilicate, CEL = lithium silicate reinforced with zirconia particles). This result is consistent with the results of Garoushi et al. [24], in which the failure load of monolithic ceramic crowns fabricated from lithium disilicate (IPS e.max CAD) was higher than zirconia-reinforced lithium silicate (Celtra Duo) after cyclic fatigue aging. LIS had smaller microcrystals and contained more glass matrices than EMA. The smaller crystals of LIS were fabricated with high-density micronization technology, which differs from what has been reported by the GC manufacturer, in which LIS has higher mechanical properties because of its smaller crystals.

Our results differ from what has been presented in the literature using ASTM standardized tests. Nassary Zadeh et al. [21] reported that cubic/tetragonal zirconia (Ceramill Zolid FX, CopraSmile, DD cubeX2, NOVAZIR MaxT, priti multidisc ZrO_2_, and StarCeram Z-Smile) had higher 4-point flexural strength than a lithium disilicate (IPS e.max Press LT A2). Holman et al. [12] reported that lithium disilicate material (IPS e.max CAD LT) had a lower flexural strength than 3Y-TZP materials (Lava Plus, 3M ESPE; Katana ML, Kuraray), a 4Y-PSZ material (Katana STML, Kuraray), and 5Y-PSZ materials (Katana STML, Kuraray; Lava Esthetic, 3M ESPE). Kwon et al. [13] reported that the flexural strength of lithium disilicate (e.max LT) = 450 ± 53 MPa was lower than that of 3Y-TZP (Katana HT) = 1194 ± 111 MPa and 5Y-TZP (Katana UTML) = 688 ± 159 MPa. The flexural tests used in these studies differ from the CTC and layered biaxial disc tests used presently.

In the present study, an occlusal thickness of 0.8 mm at the central fossae of zirconia crowns was fabricated without thermocycling, producing a range of failure loads between 3186–4260 N. Al Mortadi et al. [39] reported that there was no difference in fracture resistance between 0.7 mm and 1.1 mm for zirconia crowns. Zimmermann et al. [16] reported a failure load of 3679 N with thermocycling at a thickness of 1.5 mm for monolithic zirconia crowns (inCoris TZI C). Presently, 0.8 mm was used for glass-ceramics, which contrasts with the manufacturer’s recommendations (1.5 mm). However, Chen et al. [40] reported that minimal thickness e.max crowns (0.7 mm) did not demonstrate a significantly different fracture resistance compared to traditional thickness crowns (1.5 mm).

The TRI and JUV crowns displayed the highest failure loads. TRI crowns had a higher average failure load than JUV and PEK crowns, likely due to the presence of glass fibers as dictated by manufacturer data. Abhay et al. [41] reported that PEEK (JUV) offers good color stability, minimal abrasion, and stress modulation through plastic deformation. Aldhuwayhi et al. [31] reported that JUV crowns have a higher fracture resistance than IPS e.max^®^CAD crowns by an average of 1381 N, which is similar to our findings in JUV crowns were higher than e.max^®^Press crowns by an average of 1234 N.

The present study used the CTC test specifically to determine which cubic containing zirconia, with different yttria concentrations (3Y-, 4Y-, 5Y-PSZ), are closer to the mechanical properties of different glass-ceramics and polymers. We recognize, however, that the CTC test may be considered unreliable because, as a quasi-static test, it does not replicate the repetitive and multi-directional clinical loading of real restorations. Furthermore, higher failure loads can be attained for thicker restorations [15,16], and supported abutments with a higher modulus of elasticity [40,42]. Failure loads can further be affected by luting cement (modulus of elasticity and thickness), and the irregular geometry of a crown makes it difficult to determine the effect of specimen thickness. Additionally, the CTC test is not a standardized test, such as those recommended by ASTM and ISO, with their standardized dimensions of bar and disc geometries, polishing methods and loading apparatuses, where neither supporting abutments nor luting cements are needed.

Previous studies [30,35] presented the trilayered disc test as an alternative CTC and clinical tests. Our specimen design of trilayered disc test was replicated exactly from previous studies [30,35]. When compared with the CTC test among the three classes of materials, the results did not correlate. The failure load of crowns was generally higher than that of the corresponding discs (except PEK). The two test configurations in the present study replicated other studies (crowns used by Schriwer et al. [36] discs used by de Kok et al. [30]); therefore, the loading piston diameter (crowns = 11.37 mm, discs = 4.9 mm diameter), shape (crowns = round ended to accommodate irregular occlusal surface, discs = flat ended to accommodate flat surface), and specimen support area/ratio in the crowns and discs were different. Additionally, a polyethylene sheet was used between the steel piston and crown to distribute the stress because the loading occlusal surface of the crowns was irregular, while it was not used on discs because the surface was perfectly flat. Furthermore, the supporting epoxy resin for the trilayer design was machined from an epoxy plate to replicate de Kok et al.’s study [30], while crowns were fabricated from powder and liquid. Therefore, one may conclude that the new tri-layered disc design of specimens should not be considered an alternative to the CTC test, and the hypothesis that the failure load of crown specimens would correlate with the failure load of the disc-shaped specimens was rejected.

In the present study, artificial epoxy resin abutments were selected for securing the crowns to have a standardized abutment shape for all the specimens. We admit that the die spacer thicknesses were different between the ceramics (zirconia and glass-ceramics) and polymers to comply with the different manufacturer instructions. The crown specimens were not polished because there is no standardized polishing protocol for the occlusal surface of crowns, and the standard polishing materials are different between the different material groups (zirconia, glass-ceramic, polymer). This may have affected the measured failure loads. Other limitations of the present study include the lack of aging and fatigue testing. Lastly, the glass-materials were fabricated through a press procedure while the remaining were CAD-CAM milled, which may affect the resultant material behaviors and influence test outcomes.

## 5. Conclusions

Within the limitations of this in vitro study, the following conclusions may be drawn:

1. The crunch the crown tests revealed that the Katana UTML (3Y-PSZ) crowns had significantly lower failure loads than Cercon XT (4Y-PSZ) and BruxZir Anterior (5Y-PSZ) crowns. The IPS e.max Press specimens produced similar failure loads as zirconia crowns with Cercon XT (4Y-PSZ), and BruxZir Anterior (5Y-PSZ) but were stronger than the Katana UTML (3Y-PSZ) specimens.
2. Two polymers (Trilor and Juvora) produced the highest failure loads in both the crunch the crown and layered disc tests.
3. The failure load of the trilayer disc specimens did not correlate with the failure load of the respective crown specimens for the zirconia, glass-ceramic and polymeric materials.

## Figures and Tables

**Table 1:** The abbreviation, manufacturer, color, class, modulus of elasticity, and Poisson’s ratio of the test materials.

Test Materials	Abbreviation	Manufacturer	Color	Class	Young’s Modulus (GPa)	Poisson’s Ratio
BruxZir Anterior	BRU	Glidewell Dental Lab, Newport Beach, CA, USA	A2	Yttria-partially stabilized zirconia (Y-PSZ)	210	0.25
Katana UTML	KAT	Kuraray Noritake Dental Inc., Tokyo, Japan	A2	Yttria-partially stabilized zirconia (Y-PSZ)	210	0.25
Cercon XT	CER	Dentsply Sirona Prosthetics, York, PA	A2	Yttria-partially stabilized zirconia (Y-PSZ)	210	0.25
Celtra Press	CEL	Dentsply Sirona Prosthetics, York, PA, USA	LT shade A2	Zirconia-reinforced lithium silicate (ZLS) *	70	0.22
IPS e.max Press	EMA	Ivoclar Vivadent AG, Schaan, Liechtenstein	LT shade A2	Lithium disilicate (LS2) glass-ceramic	95	0.22
Lisi Press	LIS	GC Corporation, Tokyo, Japan	LT-A	Lithium disilicate based glass-ceramic	95	0.22
Trilor	TRI	Bioloren S.r.l., Saronno (Varese), Italy	White	Glass fiber-reinforced composite resin	26	0.25
Juvora	JUV	JUVORA Dental, Lancashire, UK	Natural	PEEK	4	0.36
Pekkton	PEK	Cendres + Métaux SA, Biel-Bienne, Switzerland	Ivory	PEKK	5	0.38
Epoxy Resin for crown	Epoxy	Die Epoxy Type 8000, American Dental Supply, Inc. Allentown, PA, USA	Gray	Epoxy resin	4	0.3
Epoxy Resin for disc	Epoxy	Epoxydplatte, Carbotec GmbH& Co. KG, Aachen, Germany	Gray	Epoxy resin	4	0.3
Metal ring	Metal	ND	Black	Steel	195	0.3

* Zirconia reinforced to increase the mechanical properties of the glass.

**Table 2:** The protocol for the “intaglio” surface treatments of the crowns and epoxy resin abutments.

Zirconia Crowns	Glass-Ceramic Crowns	Polymeric Crowns	Epoxy Resin Abutments
Alumina powder was blasted (50 µm for 10 s, with 2.5 bar pressure at a distance of approximately 10 mm with oscillatory movements), then cleaned and dried ultrasonically.	Hydrofluoric acid with 9.6% concentration was applied for 20 s, then rinsed with water and dried.	Alumina powder was blasted (50 µm for 10 s, with 2.5 bar pressure at a distance of approximately 10 mm with oscillatory movements), then etched with 40% phosphoric acid for 30 s to clean the surface, rinsed with water, and dried.	Alumina powder was blasted (50 µm for 10 s, with 2.5 bar pressure at a distance of approximately 10 mm with oscillatory movements), then etched with 40% phosphoric acid for 30 s to clean the surface, rinsed with water, and dried.

## Data Availability

The data presented in this study are available on request from the corresponding author.
